# The Fundamental Approach of the Digital Twin Application in Railway Turnouts with Innovative Monitoring of Weather Conditions

**DOI:** 10.3390/s21175757

**Published:** 2021-08-26

**Authors:** Arkadiusz Kampczyk, Katarzyna Dybeł

**Affiliations:** Department of Engineering Surveying and Civil Engineering, Faculty of Mining Surveying and Environmental Engineering, AGH University of Science and Technology, al. A. Mickiewicza 30, 30-059 Krakow, Poland; kdybel@agh.edu.pl

**Keywords:** railway safety, turnouts monitoring system, turnouts condition, digital twins in turnouts, second difference rail temperatures indicator, temperature gradient CWR, measurement, sensors, instrument

## Abstract

Improving railway safety depends heavily on the reliability of railway turnouts. The realization of effective, reliable and continuous observations for the spatial analysis and evaluation of the technical condition of railway turnouts is one of the factors affecting safety in railway traffic. The mode and scope of monitoring changes in geometric parameters of railway turnouts with associated indicators needs improvement. The application of digital twins to railway turnouts requires the inclusion of fundamental data indicating their condition along with innovative monitoring of weather conditions. This paper presents an innovative solution for monitoring the status of temperature and other atmospheric conditions. A UbiBot WS1 WIFI wireless temperature logger was used, with an external DS18B20 temperature sensor integrated into an S49 (49E1)-type rail as T_szyn_ WS1 WIFI. Measurements were made between January and May (winter/spring) at fixed time intervals and at the same measurement point. The aim of the research is to present elements of a fundamental approach of applying digital twins to railway turnouts requiring the consideration and demonstration of rail temperature conditions as a component in the data acquisition of railway turnout condition data and other constituent atmospheric conditions through an innovative solution. The research showed that the presented innovative solution is an effective support for the application of digital twins to railway turnouts and ongoing surveying and diagnostic work of other elements of rail transport infrastructure. The applicability of the T_gCWRII_ second temperature difference indicator in the monitoring of railway turnouts was also confirmed.

## 1. Introduction

The procedures in force for conducting surveying and diagnostic monitoring of the state of railway turnouts (railway switches) take into account current and modern technological solutions with the use of direct and indirect measurement methods. Unfortunately, there is still a lack of effective, continuous observation of turnouts. Railway turnouts and diamond crossings occurring on railway lines and in stations, especially marshalling yards, are bottlenecks that limit the capacity of the entire railway network. Turnouts, due to their design and geometry, enforce speed limits, which in turn affect capacity constraints. Railway switches operate in harsh environmental conditions. However, their reliability requirements are high due to safety and economic factors [1]. Turnouts are an expensive and critical feature of the railway system, as they are exposed to adverse operating loads as compared to a simple railway track and, therefore, require regular maintenance [2]. Turnouts are a critical element in the condition of railway infrastructure, being a component of turnout routes [3,4].

The functionality of railway switches and crossings (S&C) comes at a cost, as load-inducing rail discontinuities in the switch and crossing panels cause much larger degradation rates for S&C compared to the regular track [5]. Milošević et al. [5] note that railway managers are interested in developing solutions to enable reliable and flexible maintenance of S&C through remote monitoring of their condition. In [6], Marx states that professional turnout maintenance requires specialist knowledge. Complex and intricate turnout systems that include, among other things, slip turnout (double and outside slip turnout) occupy a special position in the field. Knowledge of these systems already resembles a kind of secret science.

The need to ensure the correct technical condition of turnouts and their components forces an openness to scientific research and the use of modern technological solutions. Digital twin provides a virtual representation that serves as a real-time digital equivalent of a physical object or process. Regarding the concept of using digital twins in the monitoring of railway turnouts; digital twins can provide a dynamic model of railway turnouts, with a reflection of the turnouts system and the process that exists in the real world. Identical to the physical world, there are interactions between digital objects that will mirror each other.

Kaewunruen and Lian in [7] conclude that the digital twin of a railway turnout in 3D embraces the time schedule, cost and sustainability across the whole life cycle. The use of BIM (Building Information Modeling) for railway turnout systems has the potential to improve the overall information flow of a turnout’s planning and design, manufacturing preassembly and logistics, construction and installation, operation and management, and its demolition, thereby achieving better project performance and quality [7]. As a complete information model, a digital twin integrates the information of a project from different stages of the life cycle into a model in order to facilitate better asset management and communicate through data visualizations with participants [8].

The evolution of the approaches applied to rail systems’ condition monitoring has followed manual maintenance, through methods connected to the application of sensors, up to the currently discussed methods and techniques focused on the mutual use of automation, data processing and exchange [9]. At present, the application of digital twins in the construction industry has gained significant momentum and the industry has gradually entered the information age [10]. Kaewunruen et al. [10] demonstrated the unprecedented application of digital twins to sustainability and vulnerability assessments, which can enable the next generation risk-based inspection and maintenance frameworks. Digital twin technology has the potential to transform the construction industry and provide responses to some of its challenges [11,12,13].

Digital twin application in railway turnouts requires the inclusion of fundamental data indicating their condition along with the innovative monitoring of weather conditions. In this research, the authors demonstrated the basic data that the digital twin should provide along with a special focus on atmospheric conditions.

The aim of the research was to present the elements of a fundamental approach of applying digital twins to railway turnouts requiring the consideration and demonstration of rail temperature conditions as a component in the acquisition of railway turnout condition data and other constituent atmospheric conditions through an innovative solution. A novel solution for monitoring the status of temperature and other atmospheric conditions using sensors and S49 (49E1)-type rail is demonstrated. The developed solution represents the measurement of temperature inside the rail head, humidity, ambient temperature and ambient light. For this purpose, a wireless temperature logger UbiBot WS1 WIFI was used, equipped with an external temperature sensor DS18B20 integrated with a rail type S49 (49E1)—named as T_szyn_ WS1 WIFI. The conducted research confirmed that the innovative T_szyn_ WS1 WIFI solution provides the possibility of locating it in various critical points of the railway infrastructure. At the same time, it represents a significant addition to the fundamental approach of using digital twins in railway turnouts. Acceptance was also made of the use of the T_gCWRII_ indicator of the second temperature difference in the monitoring of railway turnouts. The innovative solution is a complementary element to the fundamental approach of using digital twins in railway turnouts and Continuous Welded Rail (CWR).

## 2. Materials and Methods

### 2.1. Digital Twins in Basic Monitoring of Railway Turnouts

The digital twin in railway infrastructure should be a digital representation of the components of the infrastructure, reflecting the 3D geometry of the physical components and the current non-graphical information about these components that is used in exploitation. Powered by measurement data from surveying and diagnostics, the digital twin can support operators and infrastructure managers in their decisions by providing an analysis of the effects of different hypothetical scenarios and allowing the best, most effective solutions to be selected. Sysyn et al. [14] note that the recent identification and evaluation of the sleeper support conditions using track-side and on-board monitoring methods can help plan prevention activities to avoid or delay the development of local instabilities such as ballast breakdown, white spots and subgrade defects, etc. Németh and Fischer, in [15], have formulated recommendations on the technical applicability and technological instructions that are useful in everyday railway operation practice on the basis of the measurements and tests carried out on rail joints in the laboratory.

Developing and supporting digital twins for railway turnout solutions will require constant updating of data collection and monitoring capabilities as well as adaptive analysis and algorithms. In later stages of development, this will require monitoring of individual components. 

It is necessary to implement technologies that provide a wide spectrum of data on the technical condition of turnouts. Digital twins in basic monitoring of railway turnouts should provide data on:

1. Geometrical position, linear and angular values of the main points of the turnout.
2. The state of the parameters and indicators at the characteristic points of the turnout:
  - track gauge in main and deviated (diverted) tracks;
  - cant (superelevation) in main and deviated tracks;
  - flangeways;
  - the second height difference (profile gradient) in main and deviated tracks;
  - the twist of the main and deviated tracks;
  - state of curvature tracks;
  - the status of the switch actuator (point machines) setting forces.
3. Current weather conditions: temperature inside the rail head (crown of the rail), humidity, ambient temperature, ambient light.
4. Second difference rail temperatures indicator in railway turnouts.
5. Quantities accompanying technical studies of turnouts:
  - blade (switch rail) distance from the stock rail (throw of switch);
  - slip of hook;
  - travel path of the slide rod.
6. Additional turnout status indicators:
  - condition of the blades in the switch, wear of the blades;
  - location change single blade and stock rail in 3 axes;
  - change of blades position in relation to each other;
  - dynamic actions—contact forces at the wheel–rail contact (elements of the turnout steel sections, especially the bow of the crossing nose (frog nose) and the wing rails (knouckle rails in turnout));
  - contact distance from the firing pin blade to the beginning of the turnout;
  - perpendicularity of the position of the rail joints in relation to the track axis;
  - perpendicularity of the rail joints of the opposite;
  - anticipated condition and degradation period of the main turnout elements;
  - analysis and assessment of the turnout geometric condition in relation to permissible deviations.
7. Exploitation methods of increasing turnout durability and safety.
8. Integration with Intelligent Transportation Systems—ITS.
9. Integration with elements of the Railway Traffic Control devices (Control Command and Signaling Equipment).

Data visualization:

- geometric position of linear and angular values of main (surveying) turnout points;
- status of parameters and indicators at turnout characteristic points;
- current weather conditions;
- second temperature difference of the rails in the turnouts;
- quantities accompanying technical studies of turnouts;
- additional turnout status indicators;
- operational methods of increasing turnouts durability and safety;
- integration with Intelligent Transportation Systems—ITS;
- integration with elements of the Railway Traffic Control devices.

Gross tonnage, presence of geometry defects, ambient temperature, segment length and rail defect presence are the most important factors for predicting the risk of service failures [16]. The fundamental approach of applying digital twins to railway turnouts requires the consideration and identification of rail temperature conditions as a component in the acquisition of turnout condition data and the ability to apply appropriate diagnostics. A novel solution for monitoring temperature status and other atmospheric conditions is a significant complement to this approach. This paper demonstrates a novel solution for monitoring the status of temperature and other atmospheric conditions using sensors and S49 (49E1) type rail. Measurements were limited to an indicator of second temperature difference (T_gCWRII_) data and current atmospheric conditions for the purpose of providing turnout condition information.

This paper presents a method for measuring the temperature inside the rail head S49 (49E1) type rail using a UbiBot WS1 WIFI wireless temperature, humidity, and illumination logger equipped with a DS18B20 external temperature sensor integrated into an S49 (49E1)-type rail (T_szyn_ WS1 WIFI). The developed solution provides data acquisition on temperature and other current atmospheric conditions. A rail thermometer with wireless logger UbiBot WS1 WIFI can be located in different places of railway infrastructure. In the conducted research, it was located outside the structure gauge (infrastructure gauge), parallel to the rail tracks of the monitored turnout. The study was conducted from January 2020 to May 2020 in Poland. Observations were made inside the rail head in hourly cycles.

The second temperature difference indicator T_gCWRII_ (temperature gradient CWR) and the average temperature value T_śr_ (T_avg._) were determined from the obtained data. The research confirmed that the presented innovative solution provides a basis for measuring, acquiring and monitoring atmospheric conditions in rail transport infrastructure. The developed method for monitoring atmospheric conditions, especially in terms of integrated and advanced sensors, offers new perspectives and introduces new approaches in this leading field, while requiring new characterization analyses to guarantee precise and accurate measurements. 

### 2.2. Measuring Instruments

**Figure 1:**
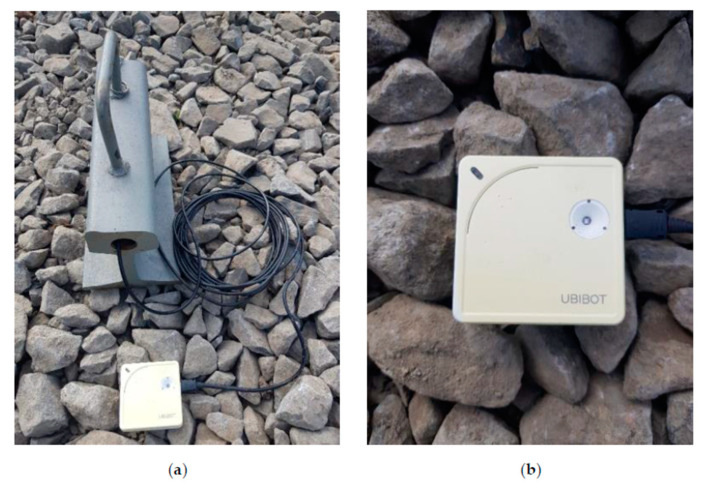
Measuring station T_szyn_ WS1 WIFI with rail type S49 (49E1) (own photograph): (**a**) electronic thermometer with probe inside crown of the rail; (**b**) wireless data logger UbiBot WS1 WIFI.

To obtain the second temperature difference data T_gCWRII_ and current weather conditions, a measuring station was established T_szyn_ WS1 WIFI containing:

- rail section ≈ 300 mm long, type S49 (49E1) (Table 1) [17], and
- electronic thermometer UbiBot WS1 (Table 2) additionally equipped with external temperature sensor DS18B20 (Table 3 and Figure 1) [18].

**Figure 2:**
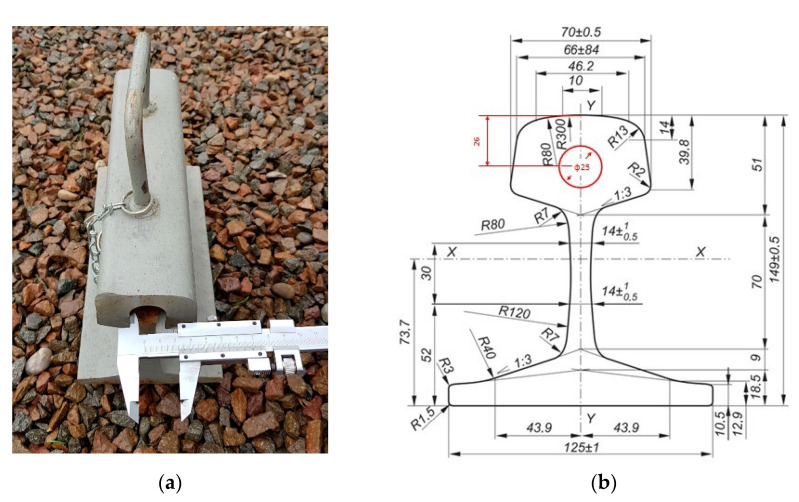
Measuring hole diameter inside the crown of the rail type S49 (49E1) (own photograph): (**a**) overhead view; (**b**) cross section.

The rail had a hole in the crown of the rail with a diameter of φ 25.0 mm and a depth of 228 mm (Figure 2).

### 2.3. Experimental Methods

**Figure 3:**
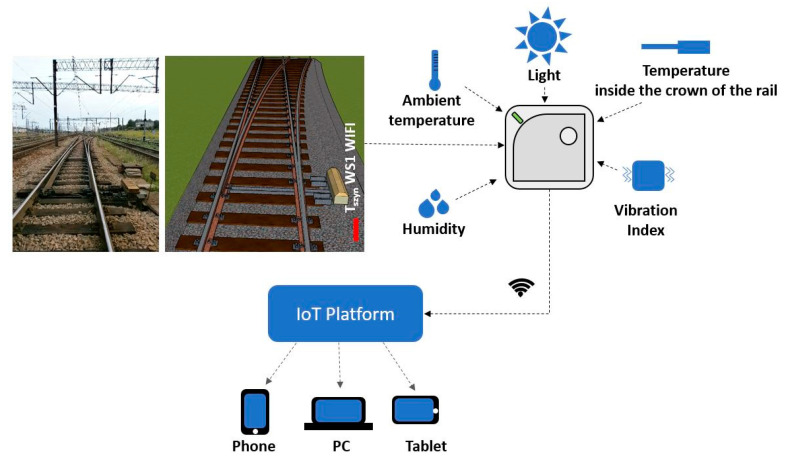
Scheme for conducting observations using measuring station T_szyn_ WS1 WIFI.

**Figure 4:**
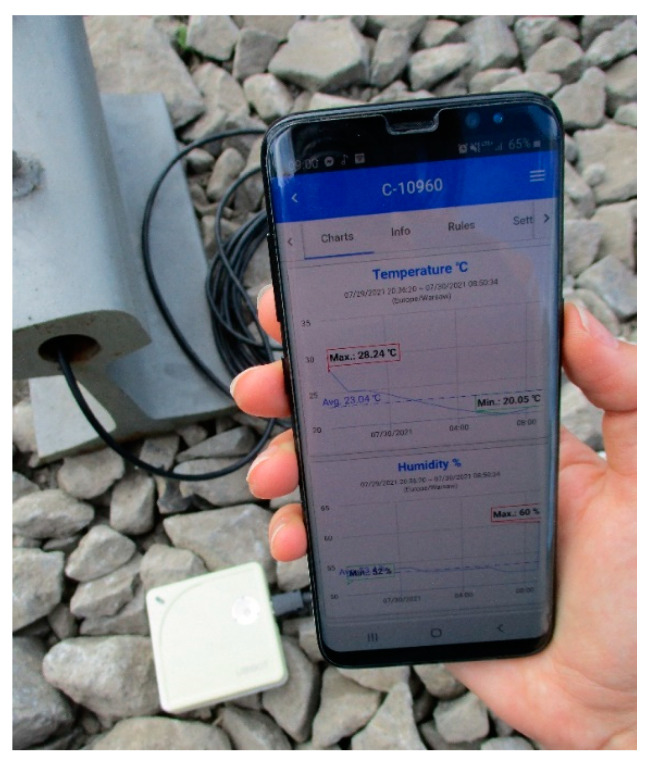
Management Console—UbiBot App.

UbiBot WS1 wireless data logger with sensors was used to acquire data on ambient temperature values, temperature values inside the rail head, humidity and ambient light. Synchronization of the performed observations with the UbiBot IoT Platform is performed wirelessly (using WIFI) in real time (Figure 3). Personalized configuration of the device is possible using the UbiBot App, which allows setting the min. cycle of observation execution and notifications that inform the user in real time if the acceptable deviation range is exceeded (Figure 4). Additionally, the UbiBot WS1 works with IFTTT (If This Then That) to create connections with other smart devices, fitting into the fundamental approach of using digital twins in railroad turnouts with innovative weather monitoring. It also provides data export in CSV (Excel) and PDF format for applications such as Continuous Welded Rail condition monitoring.

The temperature distribution of CWR rails is not uniform, which significantly complicates the precise definition of measurement principles. The selection of a representative measuring point should take into account the average deformation associated with an uneven temperature distribution [19,20]. To perform the tests, an external DS18B20 temperature sensor was placed inside the crown of the rail type S49 (49E1). A fixed measurement point was provided throughout the data collection period.

### 2.4. Reliability

The developed T_szyn_ WS1 WIFI solution comprising an S49 (49E1) rail type together with a UbiBot WS1 WIFI device having built-in memory provides for data storage in case of temporary loss of wireless connectivity to the UbiBot IoT Platform (e.g., Internet connection failure). This ensures continuity of data history. Additionally, the user can remotely check the current battery status or connect a wired power supply to the device via the built-in micro-USB port. Access to measurement data from anywhere in the world provides additional control of the device’s operation.

Integrated UbiBot WS1 WIFI device with sensors together with S49 (49E1) rail type as T_szyn_ WS1 WIFI provides data acquisition of second temperature difference indicator T_gCWRII_ and data of current weather conditions.

## 3. Results and Discussion

A proper turnout monitoring strategy can extend the service life of its various structural components. The use of appropriate measurement technology provides data for the comprehensive analysis and verification of the turnout’s condition, especially when there is an alert coming from the installed sensory sensors. 

**Figure 5:**
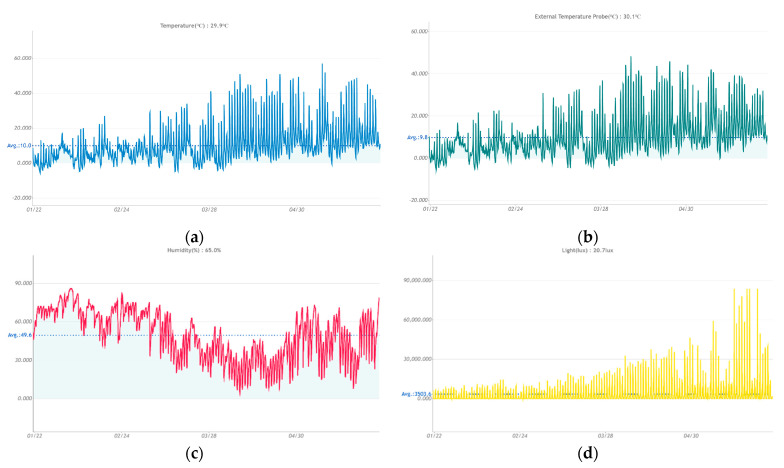
Graphical interpretation of the obtained measurement results using T_szyn_ WS1 WIFI measurement station: (**a**) ambient temperature; (**b**) temperature inside the rail head; (**c**) humidity; (**d**) ambient lighting.

During the tests, the T_szyn_ WS1 WIFI measurement station recorded data on ambient temperature, temperature inside the rail head, humidity and ambient light. Measurements were conducted from 21 January 2020 to 29 May 2020 with an hourly data recording cycle (Figure 5). A total of 5002 data points were collected from which observations made in the morning (7 a.m.), afternoon (1 p.m.) and evening (7 p.m.), respectively, were selected. This filtering of the data provided a set of observations for the same measurement epoch on each measurement day. The resulting data were used for further analysis.

From the temperature data inside the rail head, the second temperature difference indicator T_gCWRII_ was determined and is expressed by Equation (1)

(1) $$T_{\mathrm{gCWRII}}=\left(T_{2}-T_{1}\right)-\left(T_{3}-T_{2}\right)$$

where:

T_gCWRII_—indicator for second difference rail temperatures in the Continuous Welded Rail (°C)
T_1_—the temperature value in the first measuring epoch (°C)
T_2_—the temperature value in the second measurement epoch (°C)
T_3_—the temperature value in the third measuring epoch (°C)

In addition, the periodic mean temperature value from the three measurement epochs T_śr_ (2) was determined

(2) $$T_{śr}=T_{\mathrm{avg}.}=\frac{T_{1}+2T_{2}+T_{3}}{4}$$

where:

T_1_—the temperature value in the first measurement epoch (°C)
T_2_—the temperature value in the second measurement epoch (°C)
T_3_—the temperature value in the third measurement epoch (°C)

Observations were conducted at fixed time intervals and at the same measurement point during winter–spring 2020. This approach ensured that the data was recorded at moments of abrupt change, thereby increasing the likelihood of capturing possible failure states of the test object being analyzed.

**Figure 6:**
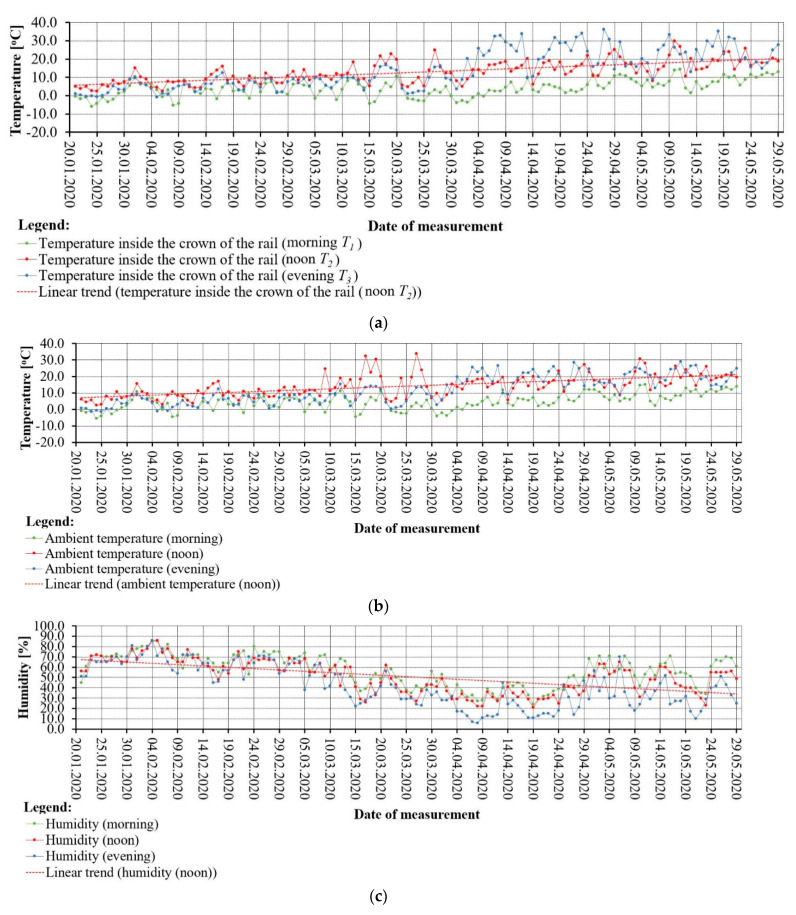

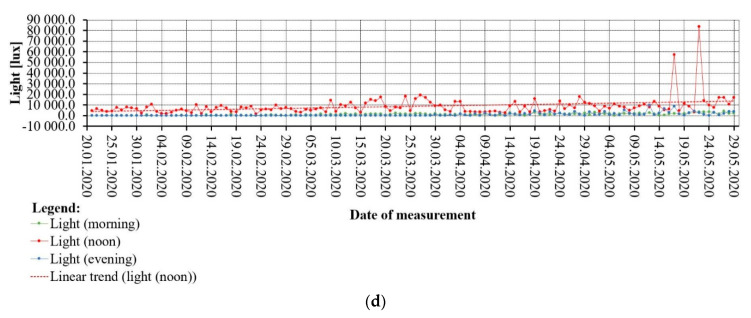
Graphical interpretation of the weather condition of the rail S49 (49E1): (**a**) temperature inside the crown of the rail, (**b**) ambient temperature, (**c**) humidity, (**d**) ambient light.

In order to determine the T_gCWRII_ indicator, measurements of the temperature values inside the rail head, taken at three measurement epochs, respectively, were used (Figure 6):

- morning T_1_,
- noon T_2_, and
- evening T_3_.

Ambient temperature, humidity and ambient light values were recorded simultaneously.

The observations obtained indicate that the rail steel is very sensitive to changes in atmospheric conditions. Table 4 shows selected data on rail temperature values from the three measurement epochs T_1_, T_2_ and T_3_ and the calculation of T_gCWRI_ and T_gCWRII_.

**Figure 7:**
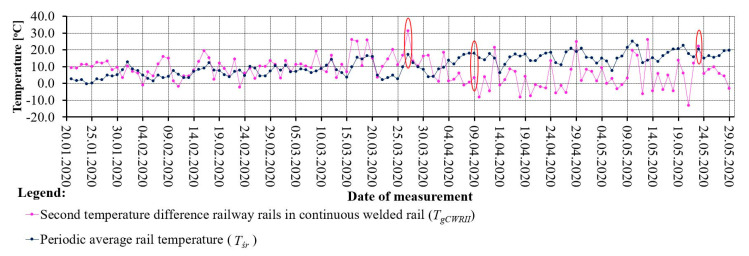
Graphical interpretation of the second rail temperature difference indicator (T_gCWRII_) and the periodic average temperature (T_śr_).

The values of the first rail temperature difference indicator T_gCWRI_ indicate the largest differences in temperature values in the afternoon. In addition, in early spring, the values of T_gCWRI_ irregularly and gradually increase, which makes it possible to notice jumps in the values of rail steel temperatures. From the values of the first rail temperature difference indicator T_gCWRI_, the values of the second rail temperature difference indicator T_gCWRII_ were calculated according to Equation (1). Figure 7 shows the values of the second temperature difference indicator T_gCWRII_ and the periodic average temperature of the T_śr_.

Analysis of T_gCWRII_ and T_śr_ values provides a quantitative estimate of the stress change over the measurement epochs. The average value of the rail temperature T_śr_ during the measurement period indicates the average thermal stress values in the rail. In cases of the simultaneous occurrence of high or low values of the periodic average temperature T_śr_ and a close to zero value of the indicator of the second temperature difference T_gCWRII_, there is a probability of failure (e.g., 9 April 2020). On the other hand, when the second temperature difference indicator T_gCWRII_ reaches values far from zero (significant temperature fluctuations) and the periodic average temperature T_śr_ is low, the probability of failure is also high (e.g., 27 March 2020). The worst condition for the rail operation is a situation in which the values of the second temperature difference indicator T_gCWRII_ and the value of the periodic average temperature value T_śr_ are far from zero (e.g., 23 May 2020). The adverse effects of other impacts that may further compound the effects of temperature changes (e.g., operational impacts) are also considered.

The possibility of failure is understood as a possibility of reaching an undesirable “critical stress–strain state”. The term “failure” is meant as the possibility of cracking (which can penetrate deep into the rail and lead to its fracture) or a buckling track.

Track buckling is a large deformation of the track. It occurs rapidly without a train load or under a passing train as a result of high longitudinal forces that exceed the resistance of the sleepers in the ballast. This is the result of large differences between the temperature in the rails and the stress-free temperature (SFT). The longitudinal forces also increase on braking sections of trains and due to creeping of the rails. Turnouts built into the CWR must have the proper operation of the individual tracks and the other components of the turnouts ensured. Failure to monitor them properly may cause malfunctions and contribute to an increase in geometrical irregularities.

The systematic creation of the database of the indicator of the second temperature difference T_gCWRII_ in the selected measurement points allows for the analyses of the frequency and degree of changes in the stress state of the continuous welded rail in a given period of time, together with other accompanying elements of the railway road. With observations from the T_szyn_ WS1 WIFI measurement station being conducted regularly in a predefined cycle (e.g., every hour), it is also possible to refine the analyses. In this case, the stage of initial data filtering is omitted or focused only on removing a small number of observations.

Many of the existing fault-diagnosis methods cannot realize real-time updating or deal with new fault types [20]. Ou, D. et al. [20] note that due to its complicated mechanical and electrical structure, exposure to the outdoor environment and the need to be frequently pulled, the turnout is more prone to failure. Conducting research on the automation of turnout condition monitoring methods is very important not only to improve the safety and reliability of railroad traffic but also to reduce maintenance costs [21,22]. The concept of using an innovative solution for monitoring the condition of temperature and other atmospheric conditions is a response to the need to automate the procedure of controlling the technical condition of turnouts.

Effective protection assures good turnout function and contributes to rail traffic efficiency and safety [23,24,25,26]. Some researchers have also proposed different incremental methods for classification problems [27]. However, many of these methods cannot be combined effectively with the field experience of workers (especially for the turnout system), and, also, some of these methods cannot update themselves, which may reduce the efficiency of the model when new samples or fault types appear since the training samples are quite limited from the very beginning [20]. In the case of applying digital twins in the monitoring of railway turnouts based on taking into account the data coming from the measurement station T_szyn_ WS1 WIFI together with the fundamental data, the created model will be dynamic and will be a reflection of the turnout system and the process existing in the real world. Additionally, digital coupling of turnout safety risks based on digital twin technology is possible [28]. In practice, digital twins can be applied in different ways leading to different designs [29,30]. 

## 4. Conclusions

The benefits of the digital twin are that it provides an easier and faster access to information, increased management efficiency using automation, a continuous analysis of component status and an effective management of turnout maintenance, which are among the weak points of the railway infrastructure. The maintenance of turnouts, and then turnout roads, is characterized by diversity in design and geometry. There are also differentiated techniques of conducting monitoring for: conventional rail systems, high-speed rail systems, passenger stations and marshalling yards. Innovative solutions, represented by digital twin railway turnouts with data from the measuring station T_szyn_ WS1 WIFI, will contribute to a reduction in the exploitation costs of selected elements of the infrastructure and will ensure control over the correctness of repairs. They will be available to infrastructure owners and managers, but also to carriers. 

The performed study method is useful for the application of digital twins mainly through its ability to provide cyclic data for analysis and verification of the turnout’s condition, especially when there is an alert coming from the installed sensory sensors. In addition, the ability to create connections with other intelligent devices, is part of the fundamental approach of using digital twins in railway turnouts.

The data from the T_szyn_ WS1 WIFI measuring station with its innovative monitoring of atmospheric conditions, which is part of the fundamental approach of the digital twin’s application in railway turnouts, make it possible to come to the appropriate diagnosis. First, the paper presented a novel solution for monitoring the condition of temperature and other atmospheric conditions for the purpose of providing information on the technical condition of a turnout. Then, an analysis method based on the second temperature difference indicator T_gCWRII_ and the periodic average temperature value T_śr_ was proposed. In line with the assumption of the need to automate data acquisition, the presented new approach fits into these requirements. In addition, the ability to personalize the measurement configuration allows the mode and cycle of observations to be adjusted accordingly. In addition, it should be noted that the proposed solution assumes the performance of running the ambient atmospheric conditions and temperature inside the rail head continuously so that the probability of capturing emergency conditions is very high.

The developed innovative solution in the form of T_szyn_ WS1 WIFI finds application not only in the system of railway turnouts but also in the monitoring of the state of the CWR together with elements of the accompanying infrastructure, engineering facilities and components of marshalling yards. Buckling tracks may also be caused by seismic movements, including in the area of railway infrastructure covering mining exploitation areas, where T_szyn_ WS1 WIFI is applied.

Based on the results of this research, it is planned to review supporting solutions that have the potential to increase the range of data recorded. The use of meteorological stations located in the vicinity of railway turnouts (near large turnout heads and marshalling yards, e.g., for comparison and forecasting purposes, will be considered.

## Figures and Tables

**Table 1:** Technical parameters of the S49 (49E1)-type rail.

Feature	Value
Cross sectional area	62.97 cm^2^
Weight per meter	49.43 kg/m
Height	149 mm
Footer width	125 mm
Width crown of the rail	67 mm
Neck thickness (web)	14 mm
Moment of inertia about the x-x axis	1819 × 10^−8^ m^4^
Moment of inertia about the y-y axis	320 × 10^−8^ m^4^

**Table 2:** Technical characteristics of the electronic thermometer type UbiBot WS1.

Type of Attribute	Value
Temperature measurement range	−20 °C to 60 °C
Humidity measurement range	10% to 90% RH
Accuracy of temperature measurement	±0.3 °C
Power supply	2 × battery AA/Micro-USB (5V/2A)
Dimensions of the device	6.5 × 6.5 × 1.7 cm
Additional equipment	external temperature sensor DS18B20

**Table 3:** Technical characteristics external temperature sensor DS18B20.

Type of Attribute	Value
Dimensions	diameter 0.6 cm, length 5 cm
Temperature measurement range	−55 °C to 125 °C
Accuracy of temperature measurement	±0.5 °C
Cable length	300 cm
Probe material	stainless steel
Cable type	circular

**Table 4:** S49 (49E1) rail temperature values of the three measurement epochs T_1_, T_2_ and T_3_ and T_gCWRI_ and T_gCWRII_ calculations.

Date of Measurement	Temperature inside the Crown of the Rail Morning T_1_ (°C)	Temperature inside the Crown of the Rail Noon T_2_ (°C)	Temperature inside the Crown of the Rail Evening T_3_ (°C)	First Difference Rail Temperatures T_gCWRI_		Second Difference Rail Temperatures Indicator T_gCWRII_ (°C)
				**T_2_ − T_1_** **=** **ΔT_I,21_ (°C)**	**T_3_ − T_2_** **=** **ΔT_I,32_ (°C)**	
10 February 2020	8.5	7.9	5.9	−0.6	−2.0	1.4
17 February 2020	4.5	16.1	12.5	11.6	−3.6	15.2
9 March 2020	−2.1	12.1	7.3	14.2	−4.8	19.0
19 March 2020	4.8	22.9	15.1	18.1	−7.8	25.9
27 March 2020	3.2	25.0	15.6	21.8	−9.4	31.2
21 April 2020	2.9	13.3	24.5	10.4	11.2	−0.8
16 May 2020	4.9	15.6	29.9	10.7	14.3	−3.6
21 May 2020	10.8	14.4	31.0	3.6	16.6	−13.0

## Data Availability

Not applicable.
